# Cytokeratin 7-negative and GATA binding protein 3-negative breast cancers: Clinicopathological features and prognostic significance

**DOI:** 10.1186/s12885-019-6295-8

**Published:** 2019-11-12

**Authors:** Shaolei Lu, Evgeny Yakirevich, Li Juan Wang, Murray B. Resnick, Yihong Wang

**Affiliations:** Department of Pathology and Laboratory Medicine, Alpert Medical School of Brown University, 593 Eddy St; APC 12, Providence, RI 02903 USA

**Keywords:** CK7, GATA3, Breast carcinoma, Immunohistochemistry

## Abstract

**Background:**

Cytokeratin 7 (CK7) and GATA binding protein 3 (GATA3) are considered as immunohistochemical hallmarks of breast cancers; however, there are breast tumors lacking these markers. Clinicopathological characterization of CK7 negative breast cancer has not been addressed previously and similar studies on GATA3 negative tumors are limited.

**Methods:**

This study included 196 consecutive cases of Nottingham Grade 3 breast cancers with 159 cases of Grade 1 and Grade 2 tumors for comparison. CK7 and GATA3 expression was correlated with patient’s age, histological type, pathological grade and stage, hormone receptor status, molecular subtype and overall survival.

**Results:**

CK7 negativity was seen in 13% of Grade 3, 9% of Grade 2, and 2% of Grade 1 cases (*P* = 0.0457). Similarly, 28% of Grade 3, 5% of Grade 2 and 2% of Grade 1 cases were GATA3 negative (*P* < 0.0001). CK7 negative tumors did not show association with other clinicopathological parameters. GATA3 negative tumors were enriched in the basal-like molecular subgroup and were associated with negative estrogen receptor (ER) and negative progesterone receptor (PR) statuses. Both CK7 and GATA3 expression showed no association with overall survival in patients with Grade 3 tumor.

**Conclusions:**

This is the first study to characterize CK7 negative breast tumors in the context of clinicopathology. Profiling the CK7 negative and GATA3 negative breast cancers helps to understand the biology of these specific tumor subgroups and may aid in their diagnosis.

## Background

Breast cancer is the most common cancer in females and is the second leading cause of cancer death in females after lung cancer [1]. It is estimated that in 2018 30% of newly diagnosed malignancies in females in the United States will be breast cancer [1]. Most of the primary breast cancers are initially diagnosed by breast biopsy following imaging studies. Cytokeratin (CK7) [2] and GATA-binding protein 3 (GATA3) [3] are two commonly used markers to confirm breast origin.

CK7 was first studied in breast tissue to differentiate luminal cells from myoepithelial cells [4]. Multiple subsequent studies have shown that CK7 was expressed in 89–98% of non-specified breast cancers [2, 5–8], in almost all medullary carcinomas [6], in the majority of micropapillary carcinoma of the breast [9] and in all mammary and extramammary Paget’s disease [10]. CK7 was expressed in 97% of triple negative breast cancer with 14.5% demonstrating less than 20% tumor cell staining [11]. Its expression was lost in most sarcomatous (23% positivity) and fibromatosis-like (17% positivity) components, but was still retained in 71% of the matrix-producing component of metaplastic breast cancer [12].

GATA3 belongs to the GATA family of zinc finger transcription factors and is involved in the development and morphogenesis of mammary glands [13]. GATA3 is considered a transcription factor maintaining the differentiation of luminal cells in the breast ducts [13]. It is one of the six genes (TP53, PIK3CA, AKT1, GATA3, CBFB and MAP3K1) with recurrent mutations in breast cancer [14]. GATA3 has been shown to be associated with the luminal subtype of breast cancer, whereas 88% of estrogen receptor (ER)-negative tumors retained GATA3 expression [15]. Its expression rate in triple-negative cancer ranged from 20.16 to 48% [16, 17] in contrast to 74.6% in apocrine type triple-negative breast cancer [18]. The majority of published studies suggest that loss of GATA3 expression is associated with worse prognosis [19]; however, this is not universally accepted [3].

There are no systematic clinicopathological studies of CK7 and GATA3 negative tumors while limited studies are available characterizing the prognostic utility of GATA3 expression in breast cancer. In the current study, we analyzed 361 cases of breast cancers (196 cases of Nottingham Grade 3 breast cancers and 159 cases of Grade 1–2 cancers) to delineate the clinicopathologic features of CK7-negative and GATA3-negative tumors and their associations with patient outcome.

## Methods

The study was performed in accordance with the ethical guidelines and approval from the Institutional Review Boards of Lifespan Health System (Rhode Island, United States).

### Patients

All cases of primary breast cancer were retrieved from the pathology archive at the Lifespan Rhode Island and The Miriam Hospitals from 2000 to 2011. The study included 196 consecutive cases of Nottingham Grade 3 and 159 cases of Nottingham Grade 1 and Grade 2 for comparison. Clinicopathological data was collected from pathological reports and medical chart review. Survival data was acquired from the Lifespan Cancer Registry and medical chart review. Chemotherapy, hormonal treatment, or radiation was considered having chemo/radiation treatment.

### Tissue microarray construction and immunohistochemical stain

Formalin-fixed paraffin-embedded tissue blocks with representative tumor areas were identified through review of corresponding hematoxylin and eosin–stained sections. Areas of interest were identified and marked on each selected block. The block was cored using a 1-mm core needle. For each case four representative cores of tumor and 1 core of adjacent non-neoplastic tissue were arrayed. The cores were transferred to the recipient “master block” using a Beecher Tissue Microarrayer (Beecher Instruments, Silver Spring, MD).

Immunohistochemical staining was performed using the Dako Autostainer Plus and EnVision Dual Link detection reagent (DAKO; Carpinteria, CA) with DAB (Dako). The following primary antibodies were used: clone OV-TL12/3 against CK7 (ready to use; DAKO) and clone L50–823 against GATA3 (1:100 dilution; Biocare Medical, Pacheco, CA). ER and PR stains were considered positive if immunostaining was seen in more than 1% of tumor nuclei. Her2 immunostain score of 3/3 or confirmed Her2/neu gene amplification were considered HER2 positive. CK7 and GATA3 staining was considered positive if 1% or more of tumor cells were stained with at least mild intensity. Immunohistochemical stains were reviewed together by two pathologists (SL and YW) to minimize inter-observer variability. Information of Nottingham grade (Grade 1, Grade 2 or Grade 3) was obtained from pathology reports.

### Statistical methods

х2 analysis was applied to evaluate associations between categorical variables. Fisher’s test was used to replace х2 analysis when appropriate. T-test was used to compare between two continuous variables. For time-to-event measures, the Kaplan–Meier method was used to estimate the empirical survival and log-rank estimates were used. For Cox proportional hazard analysis, Wald test algorithm was used. All tests were 2-sided using a *p*-value of 0.05 as threshold for statistical significance. All analyses were performed using JMP Pro 14 (SAS, Cary, NC). It was a large dataset of over 300 cases. In a small number of cases, part of clinical or staining information was not available. Minor discrepancies in case numbers were present; however, they did not affect percentages and conclusions.

## Results

### Clinicopathological features of CK7 negative and GATA3 negative breast cancers

**Fig. 1:**
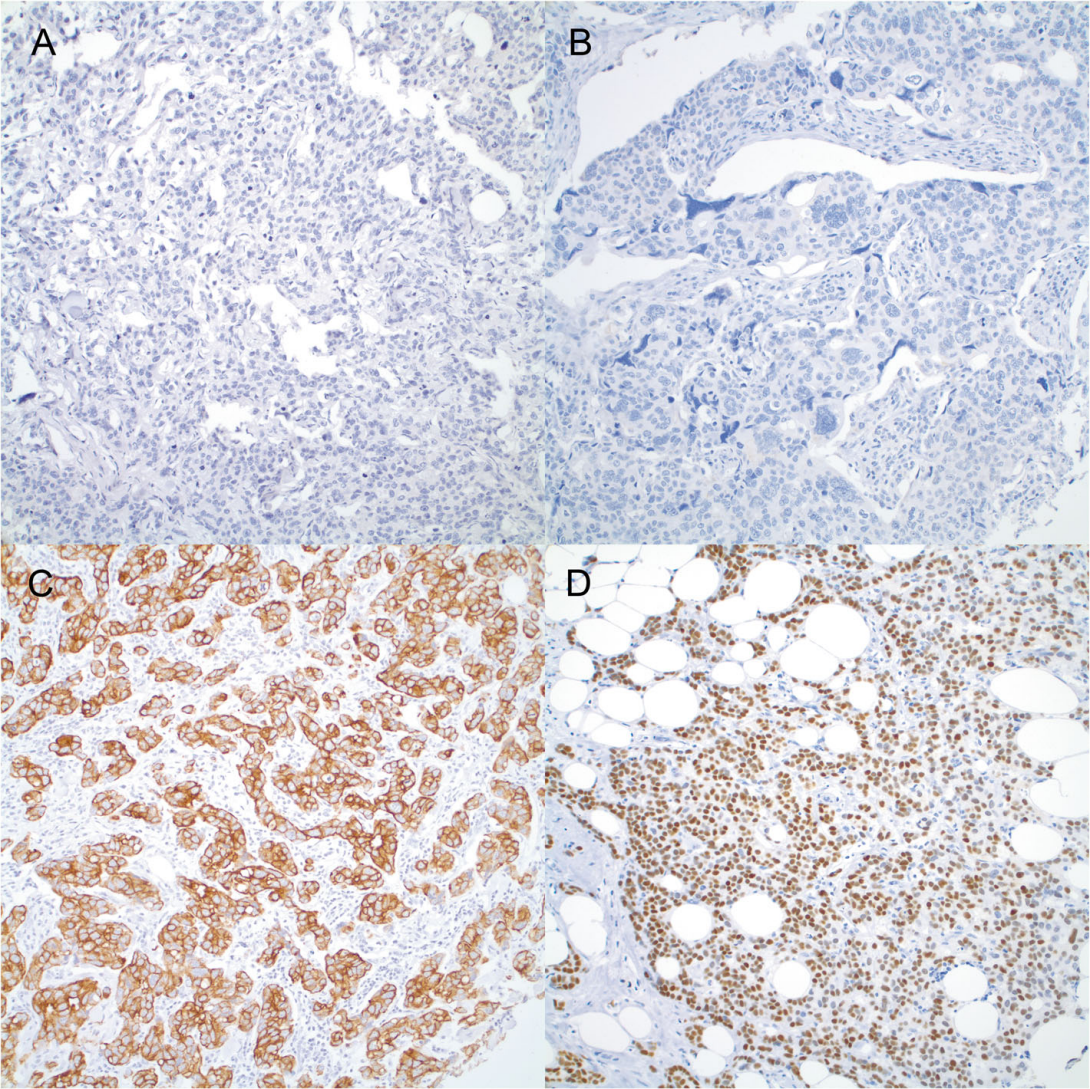
Representative breast cancers of CK7 negative (**a**), GATA3 negative (**b**), CK7 positive (**c**), and GATA3 positive tumors (**d**)

Representative tumors with strong cytoplasmic staining of CK7 and nuclear staining of GATA3 are shown in Fig. 1. Thirteen percent (13%) of Nottingham Grade 3 (G3) tumors were negative for CK7, while the negative rates were 9 and 2% for Grade 2 and Grade 1 tumors, respectively (*P* = 0.0457) (Table 1). The negative rate for GATA3 was as high as 28% in Grade 3 tumors, while only 5 and 2% of Grade 2 and Grade 1 tumors were GATA3 negative (*P* < 0.0001) (Table 2). After cases were stratified into two groups (Grade 1 and Grade 2 vs. Grade 3), patients with CK7 negative and GATA3 negative tumors had no age difference from those with CK7 and GATA3 positive tumors. No significant association was seen between CK7 and GATA3 expression, pTNM stages and Chemo/radiation treatment (Tables 1 and 2).

**Table 1:** Clinicopathological features of CK7 negative breast cancers

Nottingham grade	Low-grade (Grade 1 and 2)			High-grade (Grade 3)		
Ck7 expression	Negative	Positive	*P*-value	Negative	Positive	*P*-value
Age^a^ (N)	66 ± 4.4 (11)	63.4 ± 1.2 (142)	0.5615	62 ± 3.3 (25)	61 ± 1.2 (168)	0.7941
Nottingham grade						*0.0457*
1	1 (2%)	45 (98%)				
2	10 (9%)	97 (91%)				
3				25 (13%)	168 (87%)	
pT stage			0.8569			0.3657
1	8 (7%)	103 (93%)		11 (11%)	87 (89%)	
2	3 (9%)	30 (91%)		9 (13%)	63 (88%)	
3	0	5 (100%)		2 (14%)	12 (86%)	
4	0	4 (100%)		2 (33%)	4 (67%)	
pN stage			0.1377			0.7870
0	8 (9%)	82 (91%)		15 (15%)	85 (85%)	
1	0	35 (100%)		5 (11%)	42 (89%)	
2	0	6 (100%)		1 (8%)	12 (92%)	
3	1 (20%)	4 (80%)		1 (20%)	4 (80%)	
X	2 (12%)	15 (88%)		2 (8%)	22 (92%)	
pM stage			0.0738			0.4031
0	6 (6%)	102 (95%)		16 (13%)	106 (87%)	
1	0	0		0	2 (100%)	
x	4 (17%)	19 (83%)		2 (5%)	37 (95%)	
Chemo/radiation therapy			0.2462			0.4921
Yes	6 (6%)	98 (94%)		15 (12%)	115 (88%)	
No	4 (12%)	29 (88%)		7 (16%)	38 (84%)	

^a^Age and tumor size: mean ± SEM (standard error of mean)

**Table 2:** Clinicopathological features of GATA3 negative breast cancers

Nottingham grade	Low-grade (Grade 1 and Grade 2)			High-grade (Grade 3)		
Gata-3 expression	Negative	Positive	*P*-value	Negative	Positive	*P*-value
Age^a^ (N)	69 ± 6.7 (7)	64 ± 1.1 (152)	0.3550	57 ± 1.8 (54)	62 ± 1.4 (142)	0.0732
Nottingham Grade						*<0.0001*
1	1 (2%)	47 (98%)				
2	6 (5%)	105 (95%)				
3				54 (28%)	142 (72%)	
pT stage			0.4673			0.2223
1	4 (3%)	112 (97%)		31 (32%)	67 (69%)	
2	3 (9%)	31 (91%)		15 (21%)	58 (79%)	
3	0	5 (100%)		6 (40%)	9 (60%)	
4	0	4 (100%)		1 (14%)	6 (86%)	
pN stage			0.4247			0.2580
0	3 (3%)	93 (97%)		32 (32%)	66 (67%)	
1	1 (3%)	33 (97%)		10 (20%)	40 (80%)	
2	1 (17%)	5 (83%)		2 (17%)	10 (83%)	
3	0	5 (100%)		0	5 (100%)	
X	2 (11%)	16 (89%)		9 (33%)	18 (67%)	
pM stage			0.6172			0.9294
0	5 (5%)	106 (95%)		36 (28%)	91 (72%)	
1	0	0		1 (33%)	2 (67%)	
x	2 (8%)	24 (92%)		12 (31%)	51 (69%)	
Chemo/radiation Therapy			0.2952			0.2785
Yes	4 (4%)	103 (96%)		39 (29%)	94 (71%)	
No	3 (8%)	33 (91%)		10 (21%)	37 (79%)	

^a^Age: mean ± SEM (standard error of mean)

Next we analyzed CK7 and GATA3 expression among the different histologic types of breast cancer. The distribution of CK7 negative tumors in ductal cancers was not associated with tumor grade (*P* = 0.7565), while more GATA3 negative tumors occurred in Grade 3 ductal cancers than those in Grade 1 and Grade 2 tumors, 27% vs. 6% (*P* < 0.0001) (Table 3). All lobular, mixed lobular and ductal, cribriform, micropapillary, mucinous, and tubular tumors in this series were CK7 and GATA3 positive (Table 3). All 10 metaplastic cancers were G3 and 30 and 70% of them were CK7 and GATA3 negative, respectively (Table 3). One apocrine cancer was positive for CK7 but negative for GATA3. (Table 3).

**Table 3:** Histologic types and molecular features of CK7 negative and GATA3 negative breast cancers

Histologic type and molecular features	CK7 negative	CK7 positive	*P-*value	GATA3 negative	GATA3 positive	*P*-value
Ductal			0.7565			<0.001
Grade 3	22 (13%)	147 (87%)		47 (27%)	126 (73%)	
Grade 1 & Grade 2	11 (12%)	83 (88%)		6 (6%)	91 (94%)	
Lobular & Mixed						
Grade 3	0	37		0	36	
Grade 1-Grade 2	0	18		0	20	
Metaplastic (all Grade 3)	3 (30%)	7 (70%)		7 (70%)	3 (30%)	
Cribriform (all Grade 1-Grade 2)	0	5		0	6	
Micropapillary (all Grade 1-Grade 2)	0	4		0	4	
Mucinous (all Grade 1-Grade 2)	0	5		0	5	
Tubular (all Grade 1-Grade 2)	0	3		0	3	
Apocrine (Grade 2)	0	1		1	0	
ER (Grade 3 only)			0.2822			*<0.0001*
Positive	14 (16%)	72 (84%)		2 (2%)	89 (98%)	
Negative	11 (10%)	95 (90%)		52 (50%)	52 (50%)	
PR (Grade 3 only)			0.0270			*<0.0001*
Positive	15 (20%)	61 (80%)		5 (6%)	74 (94%)	
Negative	10 (9%)	106 (91%)		49 (42%)	67 (58%)	
Her2 (Grade 3 only)			0.9434			0.0826
Positive	6 (13%)	39 (87%)		9 (18%)	40 (82%)	
Negative	19 (13%)	128 (87%)		45 (31%)	101 (69%)	
Subtype (Grade 3 only)			0.6154			*<0.0001*
Luminal	10 (16%)	76 (89%)		1 (2%)	63 (98%)	
Her2	6 (13%)	39 (87%)		9 (18%)	40 (82%)	
Basal-like	9 (11%)	76 (90%)		44 (54%)	38 (46%)	

### Receptor status and molecular subtype in CK7 negative and GATA3 negative grade 3 breast cancers

ER, PR, and Her2 expression were not found to be associated with CK7 expression in Grade 3 breast cancer (*P* = 0.2822, 0.0270 and 0.9434, respectively) (Table 3). About 50% of ER negative Grade 3 tumors were also GATA3 negative, in contrast to 2% of ER positive tumors being GATA3 negative (*P* < 0.0001). Similarly, 42% of PR negative Grade 3 tumors were GATA3 negative, in contrast to 6% in PR positive tumors (*P* < 0.0001). More GATA3 negative tumors were seen in Her2 negative (31%) than those in positive tumors (18%); however, the difference was not statistically significant (*P* = 0.0826) (Table 3).

Based on the expression of ER and HER2, Grade 3 tumors were grouped into luminal (ER+/HER2−), HER2 enriched (HER2+/ any ER), and basal like (ER−, HER2−) [20, 21]. CK7 negative tumors were evenly distributed in all the subtypes (*P* = 0.6154), while GATA3 negative tumors were enriched in the basal-like group (P < 0.0001) (Table 3).

### Characteristics of CK7 and GATA3 double negative breast cancers

There were 8 tumors negative for both CK7 and GATA3 (2.4% of all), including 6 ductal and 2 metaplastic tumors. Seven out of 8 (87.5%) were Grade 3 tumors; the remaining one was Grade 2. All 8 tumors were negative for both ER and PR. Only one was positive for Her2 (Table 4). Among Grade 3 tumors, 17% of ER positive and 21% PR positive tumors were seen in CK7 negative/GATA3 positive tumors, in contrast to 1% of ER positive and 45% PR positive tumors being CK7 positive/GATA3 negative (*P* < 0.0001) (Table 4). Her2 expression did not have any association with CK7/GATA3 status (*P* = 0.4027) (Table 4).

**Table 4:** Characteristics of CK7 and GATA3 double negative Grade 3 breast cancers

Features	CK7−/GATA3-	CK7−/GATA3+	CK7+/GATA3-	CK7+/GATA3+	*P*-value
Counts	8 (2.4%)	28 (85.4%)	51 (15.5%)	241 (73.5%)	
Nottingham Grade					<0.0001
1	0	1 (2.3%)	1 (2.3%)	41 (95.3%)	
2	1 (1%)	9 (8.8%)	5 (5%)	87 (85.3%)	
3	7 (3.8%)	18 (9.8%)	45 (24.5%)	113 (61.7%)	
ER (G3 only)					<0.0001
Positive	0	14 (17%)	1 (1%)	69 (82%)	
Negative	7 (7%)	4 (4%)	44 (45%)	43 (43%)	
PR (HG)					<0.0001
Positive	0	15 (21%)	4 (5%)	54 (74%)	
Negative	7 (6%)	3 (3%)	41 (38%)	58 (53%)	
Her2 (HG)					0.4027
Positive	1 (2%)	5 (12%)	7 (16%)	30 (70%)	
Negative	6 (4%)	13 (9%)	38 (27%)	92 (59%)	

### Clinical outcomes in CK7 negative and GATA3 negative tumor patients

**Fig. 2:**
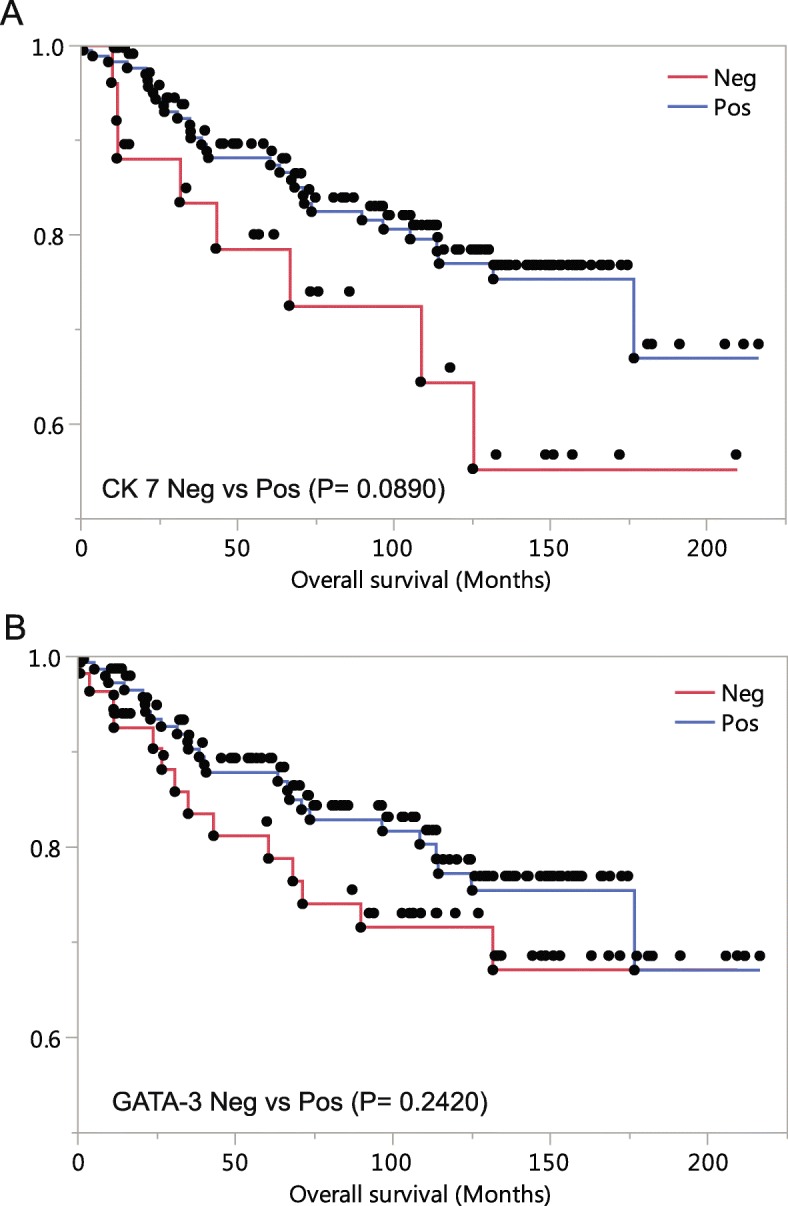
Survival analysis of CK7 negative and GATA3 negative Grade 3 breast cancer patients. **a** Overall survival in CK7 negative vs. positive cancer patients. **b** Overall survival in GATA3 negative vs. positive cancer patients

The mean follow-up time in this series was 102 months with the longest of 216 months. Patients lost to follow-up or who died of causes other than breast cancer were censored. Patients with Grade 3 tumor were included in the survival analysis. Patients with CK7 negative tumors showed worse overall survival as compared to those with CK7 positive tumors; however, the difference was not statistically significant (*P* = 0.0890) (Fig. 2a). Patients with GATA3 negative tumors initially showed worse prognosis than these with GATA3 positive tumors, but the difference narrowed toward the end of the observation (*P* = 0.2320) (Fig. 2b). Grade 3 tumors in absence of both CK7 and GATA3 showed trend of worse patient outcome; however, there was no statistical significance (*P* = 0.2014).

In univariate Cox proportional hazard analyses, Her2, pT stage, and pN stage showed significant impacts on the prognosis of patients with Grade 3 tumors (*P* = 0.0018, *P* < 0.0001, *P* = 0.0012, respectively) (Table 5). In multivariate analysis, ER, pT stage, and pN stage showed significant prognostic impact (*P* = 0.0314, *P* = 0.0143 and *P* = 0.0317, respectively). CK7 and GATA3 did not show significant prognostic impact in both univariate and multivariate analyses (Table 5).

**Table 5:** Univariate and multi-variants Cox proportional hazard analyses of CK7 and Gata3 expression in Grade 3 breast cancers

Parameter	Univariate [lower 95%, upper 95%]	*P*-value	Multi-variant [lower 95%, upper 95%]	*P*-value
Age	[−0.025386, 0.015766]	0.6547	[−0.062527, 0.0038646]	0.0858
CK7 [Pos-Neg]	[−1.386215, 0.1862815]	0.0949	[−1.512252, 0.4173403]	0.1972
Gata3 [Pos-Neg]	[−1.013242, 0.2908032]	0.2556	[−0.969737, 1.0802151]	0.9156
ER [Pos-Neg]	[−1.1454, 0.0346665]	0.0659	[−2.069892, − 0.1064]	*0.0314*
Her2 [Pos-Neg]	[−1.858073, − 0.233523]	*0.0081*	[−2.013318, 0.0923009]	0.1037
pT		*< 0.0001*		*0.0143*
T2-T1	[−0.191511, 1.129611]		[−0.643541, 1.1878657]	
T3-T2	[0.6830868, 2.4074455]		[−0.127867, 2.283623]	
T4-T3	[−1.232561, 1.0674436]		[−1.01393, 2.770524]	
pN		*0.0012*		*0.0317*
N1-N0	[−0.025245, 1.3477474]		[0.1051354, 1.9312744]	
N2-N1	[−0.767612, 1.439705]		[−3.543852, 1.1203884]	
N3-N2	[0.0520156, 2.7110952]		[−0.497292, 4.7129041]	
Nx-N3	[−2.562949, −0.230165]		[−2.618863, 0.859423]	
Chemo/radiation	[−0.742833, 0.6887183]	0.8420	[−1.389767, 0.830584]	0.5446

## Discussion

Since there are no systematic clinicopathological studies on CK7 and GATA3 negative breast tumors and limited studies characterizing the prognostic utility of GATA3 expression in breast cancer, the current study provided detailed information that is lacking in the literature.

CK7 staining is not routinely used in the primary breast cancer diagnosis except in rare cases when clinical and pathological evidence prompt the need to exclude a secondary cancer source. CK7 negative breast tumors may be diagnostically challenging in metastatic tumors and cancers of unknown origin. A comprehensive characterization of CK7 negative breast cancer may be scientifically and diagnostically useful.

In the current study, we found that as high as 13% of Grade 3 breast cancers and 30% of metaplastic cancers were CK7 negative. CK7 expression was not significantly associated with age, stage, receptor status and molecular subtype. Among the 11 histologic types included in the study, loss of CK7 expression was only seen in ductal or metaplastic tumors.

Based on Grade 3 breast cancers, survival analysis showed CK7 expression had no impact on overall outcomes (Fig. 2). Given the fact that Grade 1 and 2 tumors are more likely to retain CK7 expression and associated with better prognoses, survival analysis of breast tumors of expected grade distribution (about 34% of Grade 3 instead of 55% in this series, see below) will likely show significant association of CK7 expression loss with poor patient outcome. Indeed, if Grade 1 and Grade 2 tumors were included in the current series, a significant association were seen between worse overall survival and CK7 expression loss patients (*P* = 0.0084) (Additional file 1: Figure S1A). Loss of CK7 in the tumor could indicate initiation of a different differentiation pathway, presence of epithelial to mesenchymal transition, dedifferentiation, or enhanced tumor “stemness”. More studies should be carried out for further investigations.

As opposed to CK7 negative tumors, GATA3 negative breast cancer has gained more attention from both diagnostic pathologists and cancer biologists. The percentage of GATA3 negative breast cancers vary from study to study, ranging from 0 to 46% in ER positive tumors and from 17 to 97% in estrogen negative cancers [3]. In the current study, the negative rates were 1.7% in ER positive and 48.6% in ER negative tumors. Among the published studies in which the same antibody and the same positivity cut-off were used as in this study, GATA3 negative rates ranged from 17 to 56%, consistent with our finding of 17.1%. Reported GATA3 negative rates in metaplastic cancers ranged from 44 to 82%, which was consistent with the 70% (7 out of 10) in the current study. In addition we found that none of the lobular tumors and those with mixed lobular and ductal features was GATA3 negative, which was compatible with most published studies [3].

GATA3 is required for ER-dependent cellular processes and GATA3 and ER participate in positive feedback loops, each stimulating the expression of the other [21]. Forced expression of GATA3 in mouse mammary cancer model showed a protective effect with improved prognosis [22]. Mutations of GATA3 in breast cancer are relatively common. Based on analyses performed through cBioPortal [23, 24] on the largest publicly available breast cancer dataset, Molecular Taxonomy of Breast Cancer International Consortium (METABRIC) [25], 11.5% (250/2173) of breast tumors harbored somatic mutations of GATA3. Out of the 250 mutations, 193 (77.2%) were truncating, 52 (20.8%) were missense and 5 (2%) were inframe mutations. GATA3 mutations were more frequently seen in mixed invasive and mucinous cancers (*P* = 0.0124) and more likely to be estrogen receptor positive (*P* < 0.0001), Her2 negative (*P* = 0.0002), with lower grades (*P* < 0.0001), and with lower pT stages (Grade 1 and 2) (*P* = 0.0254) (Additional file 1: Table S1). GATA3 mutated tumors were associated with better patient outcomes (*P* = 0.0018) (Additional file1: Figure S2). Mutant GATA3 proteins mainly interfere with the DNA binding but the transactivation domains are largely intact [22]. GATA3 antibody we used (clone L50–823) was raised against peptide segment between transactivation and DNA-binding domains and presumably captures both wild-type and mutant protein versions. In the current study, GATA3 was positive in half of the ER negative tumors, consisting of 28% of all Grade 3 tumors (Table 3). Further investigations should be focused on the mechanisms how GATA3 maintains its expression without activating ER.

Published data regarding GATA3 as a prognostic marker are conflicting. Loss of GATA3 expression has been associated with unfavorable clinical outcome and worse survival [15, 26, 27]. However, no association with outcome has been observed in other studies [28]. In one study, GATA3 expression was found to be associated with favorable outcome in all the breast tumors in the study, while the association was lost when only ER positive cancers were analyzed [29]. GATA3 expression is closely associated with ER and PR expression and loss of GATA3 expression is postulated to be similar to loss of ER expression prognostically. However, our study did not see similar findings in 196 Grade 3 breast cancers. Multiple factors could contribute. First, the case number was not large enough to bear sufficient statistical power. Second, ER and GATA3 are closely related in the hormonal pathway, but they could still have distinct functions as to tumor progression. Third, in the current practice, vast majority of ER positive tumors have been treated hormonally. The treatment may impact the prognosis. Four, our survival study only included Grade 3 tumors and the adding more Grade 1 and Grade 2 tumors is likely to include more GATA3 positive tumors with better prognosis and thus with a significant prognostic difference. Indeed, repeating analysis by adding Grade 1 and Grade 2 tumors in the current series showed significant worse survival of GATA3 negative cancer patients (*P* = 0.0063) (Additional file 1: Figure S1B).

Our study is limited by a high proportion of Grade 3 breast cancer (55%) while it was estimated that only a third of breast cancer are Grade 3 [30]. A significant amount of additional work would be involved if all the concurrent Grade 1 and Grade 2 tumors were included in the study. Results that would be affected by disproportional Grade 1 and Grade 2 cases include survival analyses and association studies when markers were not evenly distributed among grades, such as ER, PR, Her2 and pTNM status. Therefore, these analyses were performed only in Grade 3 tumors except association studies that directly involved the grade.

Another possible limitation is about the proportions of each special type cancer in this study. Our study included 10 metaplastic (0.28% of all tumors), 6 cribriform (1.6%), 4 micropapillary (1.1%), 5 mucinous (1.4%), 3 tubular (0.8%) and 1 apocrine cancer (0.3%). The cribriform and tubular tumors were slightly overrepresented and mucinous and apocrine tumors were slightly underrepresented, comparing to known percentages for each special type: 0.2 to 5% for metaplastic, 0.3–0.8% for cribriform, 0.9–2% for micropapillary, 2% for mucinous, 2% for tubular and 4% for apocrine cancers [31]. However, the differences do not significantly affect the main conclusions in this study.

The mainly purpose of the study is to understand the pathobiology of the CK7 negative and GATA3 negative breast cancer. If we want to apply the findings in this study to facilitate the diagnosis of metastatic breast cancer, we need to know the CK7 and GATA3 expression in matched pairs of primary and metastasis. It certainly requires a separate study. A small portion of CK7 and/or GATA3 negative cases in our series later developed tumor metastasis; however, CK7 and GATA3 immunostains were not used to form the diagnoses of these metastases. Some of the metastases occurred before GATA3 immunostain became available. However, clinical information, microscopic morphology and other mammary gland markers were often enough to establish the diagnoses. In a pilot study, we collected 12 pairs of primary tumor and liver metastasis and 12 pairs of primary tumor and bone metastasis. There was no GATA3 expression change in primaries and liver metastases: all 10 GATA3 positive primaries retained GATA3 expression in their paired liver metastases and GATA3 expression in 2 negative primaries remained negative in the paired liver metastases. Some of the GATA3 expression was lost in bone metastasis: GATA3 expression in 8 positive primaries was only retained in 4 paired bone metastases and GATA3 expression in 4 negative primaries remained negative in the paired bone metastases. The exact reason why GATA3 expression was lost in some bone metastases is unclear. It could be true loss of expression (such as post-chemotherapy) but it is more likely to be related with tissue processing such as decalcification or sampling error due to the small size of bone biopsy. The same type of sampling error could happen to core biopsy or fine needle aspiration (FNA). Further studies are needed to explore GATA3 and CK7’s role in diagnosing metastases of special cancer types, such as metaplastic, mucinous, and apocrine tumors.

In the current series, we identified 8 cases of CK7 and GATA3 double negative breast cancer. All eight cases have been considered as primary breast cancer and pathological diagnoses were made without CK7 and/or GATA3 stains. It could have been challenging if CK7 and GATA3 immunostains were performed at the time of diagnosis. During the current study, we had retrospective chart review for these cases including any follow-up history and no non-breast primary tumor was identified. In our practice, GATA3 immunostain is routinely performed on any triple-negative cancers that do not have an in situ component. Ultimately, only history and follow-up can definitively confirm the breast origin in some cases.

## Conclusions

This is the first study that comprehensively characterized clinicopathological and prognostic features of breast cancers missing either of two canonical markers, CK7 and GATA3. There was 13% CK7 negative and 28% GATA3 negative tumors in Grade 3 breast cancer. CK7 negative tumors showed no association with most clinicopathological features and loss of CK7 expression was not associated with worse outcome in patients with Grade 3 tumors. Loss of GATA3 expression was associated with negative ER status and GATA3 negative tumors were enriched in the basal-like molecular subgroup. About half of the ER negative breast cancers retained GATA3 expression and no overall survival was seen associated with GATA3 negative Grade 3 tumors. Profiling the CK7 negative and GATA3 negative breast cancers helps to understand the biology of these specific tumor subgroups and may aid in their diagnosis.

## Supplementary information

**Additional file 1: Table S1.** Clinicopathological features of GATA3 mutated breast cancer in METABRIC dataset. **Figure S1.** Survival analysis of CK7 negative and GATA3 negative cancer patients (including Grade 1–3). A. Overall survival in CK7 negative vs. positive cancer patients. B. Overall survival in GATA3 negative vs. positive cancer patients. **Figure S2.** Survival analysis of 2173 patients/samples in the METABRIC studies based on GATA3 mutation status.

## Data Availability

Deidentified patient data from this study will not be shared publicly, but are available from the corresponding author on reasonable request.
