# Supplementary material for: Cytokeratin 7-negative and GATA binding protein 3-negative breast cancers: Clinicopathological features and prognostic significance
Source: BMC Cancer. 2019 Nov 12;19:1085. doi: 10.1186/s12885-019-6295-8 (PMC6849242; doi:10.1186/s12885-019-6295-8)
Supplement: Supplementary file 1 — Additional file 1: Table S1. Clinicopathological features of GATA3 mutated breast cancer in METABRIC dataset. Figure S1. Survival analysis of CK7 negative and GATA3 negative cancer patients (including Grade 1–3). A. Overall survival in CK7 negative vs. positive cancer patients. B. Overall survival in GATA3 negative vs. positive cancer patients. Figure S2. Survival analysis of 2173 patients/samples in the METABRIC studies based on GATA3 mutation status. [file 12885_2019_6295_MOESM1_ESM.docx]

**Table S1** Clinicopathological features of GATA3 mutated breast cancer in METABRIC dataset

| Parameters | GATA3 mutated | GATA3 wildtype | P-value |
| --- | --- | --- | --- |
| Breast cancer (totally 2173 cases) | 250 (11.5%) | 1923 (88.5%) |  |
| Mutation types  Truncating  Missense  Inframe | 193 (77.2%)  52 (20.8%)  5 (2%) |  |  |
| Cancer Type  Invasive ductal  Invasive lobular  Mixed invasive and mucinous  Mixed invasive ductal and lobular  Metaplastic | 183 (11%)  13 (7.5%)  7 (28%)  31 (14%)  0 | 1477 (89%)  159 (92.5%)  18 (72%)  187 (86%)  2 (100%) | 0.0124 |
| Estrogen receptor  Positive  Negative | 235 (15%)  5 (1%) | 1382 (85%)  518 (99%) | < 0.0001 |
| Her2  Positive  Negative | 11 (4%)  219 (13%) | 236 (96%)  1514 (87%) | 0.0002 |
| Histologic grade  Grade 1  Grade 2  Grade 3 | 35 (20%)  117 (14%)  77 (7%) | 139 (80%)  734 (86%)  970 (97%) | < 0.0001 |
| pT stage  T1  T2  T3  T4 | 70 (13%)  98 (11%)  7 (6%)  3 (30%) | 458 (87%)  773 (89%)  119 (94%)  7 (70%) | 0.0254 |

**Figure S1**. Survival analysis of CK7 negative and GATA3 negative cancer patients (including Grade 1-3). A. Overall survival in CK7 negative vs. positive cancer patients. B. Overall survival in GATA3 negative vs. positive cancer patients.

**Figure S2**. Survival analysis of 2173 patients/samples in the METABRIC studies based on GATA3 mutation status.
